# Military Personals

**Published:** 1917-11

**Authors:** 


### MILITARY PERSONALS

Lt. F. C. Robbins of Hornell has been ordered to Columbia, S. C.

Capt. G. B. Campbell of Utica has been ordered to Little Rock, Ark., for duty.

**Military Service.** (Note: Both the Journal and the County Societies have experienced the utmost difficulty in obtaining full notes of this nature. We can not even ask the Surgeon General or the Adjutant General to sort over an index of many thousand names. There are various more or less practical benefits that physicians in military service will obtain from having the fact of their absence or of awaiting orders, known to the profession. Among these benefits are commutation of society dues, reading matter which most journals would probably contribute or secure, protection of practices, etc. Anyhow, just as a matter of friendly interest, we want to make these notes as full as possible. Please notify us of omissions).

---

Dr. G. K. Smith of Syracuse has been appointed Lt. and ordered to Camp Mills, Garden City, L. I.

---

Dr. W. E. Muns, City Bacteriologist, of Syracuse, has been commissioned Lt. and ordered to Washington.

---

Dr. Edward J. Wynkoop of Syracuse has been commissioned Capt.

---

Dr. R. L. Crockett, Mayor of Oneida, has been ordered to Ft. Meade.

---

The following medical officers of what was the 74th Reg., N. G. N. Y., have been assigned to special duty as follows: Capt. Arthur C. Schaefer, attached to Div. Surgeon's staff for sanitary duty; Lt. Harry Girvin, to a field hospital; Lt. Harry Nealon to the 1st Field Hospital Co.

---

Lts. A. A. Gartner and W. L. Machemer of Buffalo have been assigned to duty with Base Hospital No. 23.

---

Lt. C. A. Lawler of Salamanca has been ordered to Hoboken for duty.

---

Drs. A. C. Abbott and Scott R. Fisher of Syracuse, members of the staff of the Crouse-Irving Hospital, have been appointed Lts. and ordered to the Army Medical School at Washington.

---

Dr. George S. Skiff, Buffalo 1887, of Gainesville has been appointed to the Medical Corps of the Army. His position as Health Officer of Gainesville has been assumed by Dr. L. H. Humphrey of Silver Springs.

---

Dr. George F. Cott of Buffalo, President-nominee of the Erie Co. Medical Society, has been commissioned Captain and is examining applicants for the Aviation Corps.

---

Dr. Wilfred H. Baines, Lt. M. R. C., was ordered from Ft. Benj. Harrison to duty in a French Hospital, Sept. 20.

Dr. T. B. Bond of Cuba, left for Ft. Oglethorpe, Ga., Oct. 5. (The name of the camp reminds us to remind our readers that if they are not already well informed on the colonial wars of America, this part of history is extremely interesting, particularly as showing how much better prepared this country was for the Revolution than for succeeding wars—relatively to military standards of different centuries).

---

Lt. Edward Hanes of Rochester has been ordered to Washington Barracks to make examinations in his specialty.

---

Lt. F. C. Devendorf of Utica has been ordered to Camp McClelland, Anniston, Ala., to make examinations in his specialty.

---

Capt. Robert King, of the Buffalo State Hospital staff, has been ordered to Camp Wheeler, Macon, Ga., to make examinations in his specialty.

---

The following have been ordered to Ft. Benj. Harrison, Ind., for instruction: Major Frank A. Walder, Lockport; Capt. Otto C. J. von Renner, Buffalo; Capt. Walter H. Vosburg, Dunkirk; Lt. Joseph J. Duuigan, Auburn; Paul E. Betowski, Bath; Arthur E. McCarthy, Buffalo; Windsor R. Smith, Buffalo; Victor A. Tyrasinski, Buffalo; Henry S. Edmunds, Cassadaga; Hall G. Van Vlack, Forestville; Wm. M. Sill, Jamestown; Bernard S. Strait, Penn Yan; Roy L. Marshall and Le Clare Stuart, Rome; Jas. A. Taggert and Geo. B. Ubel, Salamanca.

---

Lt. Arthur G. Pilch of Rochester has been ordered to Ft. Oglethorpe for instruction.

---

Major Chas. W. Hennington of Rochester, our Associate Editor, has been ordered to the Post Graduate Hospital, N. Y., and thence to Boston, for instruction in orthopaedics.

---

Major Edward S. Van Duyn of Syracuse, having completed his special service for the War Dept. in that city, has been returned to the inactive list of the M. R. C.

---

Lt. John A. Conley of Penn Yan, has been ordered to return to his home, after having completed his course of instruction at Plattsburg.

---

Dr. Grover W. Wende of Buffalo, our Associate Editor, has been called to Washington by the Surgeon-General to assist in planning a campaign against venereal diseases. He expects to return to Buffalo toward the end of November.

---

Capt. Chas. W. Selover of Stanley, and Lt. Benj. H. Dike of Rochester, have been ordered to Newport News for duty with the 302d Stevedore Reg.

---

Lt. Maurice D. Barnette of Watertown, has been ordered to the Walter Reed General Hospital, Takoma Park, D. C., for duty as a surgical assistant.

---

Lt. W. L. Weeden of Clifton Springs has been ordered to duty at Camp Hancock, Ga.

---

Lts. Victor A. Tyrasinski, Buffalo; Lloyd W. Ballantine and John J. Corbett, Syracuse, have been ordered to Camp Taylor, Louisville, Ky., for duty in connection with sanitary trains.

---

Capt. John R. Bradley of Rochester, Lts. H. W. Culbertson, Buffalo, Theron B. Bond, Cuba, have been ordered to Ft. Oglethorpe for instruction.

---

Lt. T. G. Tousey of Rochester has been ordered to Ft. Ontario, N. Y., for duty with Field Hospital and Ambulance Companies.

---

Lt. P. J. Barone, Buffalo, has been ordered to Garden City, L. I., for duty.

---

Lt. F. J. Garlick of Rochester has been ordered to the Neurologic School, U. of P., Philadelphia, for intensive training in brain surgery.

---

Dr. F. C. Purcell of Jamestown, has been commissioned 1st Lt. and surgeon in Co. E, 74th Reg. for state service.

---

Lt. H. B. Wertz of Buffalo, arrived in England about the middle of Oct.

---

Dr. Karl F. Eschelman of Buffalo has been appointed surgeon to the 74th Battalion, for state service.

---

Capt. Frederick T. Van Beuren of N. Y. City, has been ordered to special duty in Buffalo, Rochester and Utica, in connection with the M. R. C.

Lt. Abraham Lebendig of Rochester has been ordered to Cornell Medical College, N. Y. City, for instruction in military roentgenology.

---

Capt. James E. Holden of Collins has been ordered to duty at Ft. Adams, R. I.

---

Lts. Jerome A. Murphy and Alfred H. Vogt of Buffalo, and Ernest G. Gilmore of Johnstown have been ordered to Ft. Oglethorpe, Ga., for instruction.

---

Capt. Ira A. Allen of Ludlowville has been ordered to Ft. Slocum, N. Y., for duty.

---

Lt. Scott B. Fisher of Syracuse, has been ordered to Mineola, L. I., for duty in connection with the Aviation School.

